# The influence of green tea, caffeine, conjugated linoleic acid and branched chain amino acids on body composition and abdominal fat in overweight and obese individuals

**DOI:** 10.1186/1550-2783-10-S1-P19

**Published:** 2013-12-06

**Authors:** Michael J Ormsbee, Shweta R Rawal, Amber W Kinsey, Takudzwa A Madzima, D David Thomas, Nicholas Fisher, Marcus E Elam, Maria T Spicer

**Affiliations:** 1 Department of Nutrition, Food, and Exercise Sciences, The Florida State University, Tallahassee, FL. USA

## Background

Green tea, caffeine, conjugated linoleic acid (CLA), and branched chain amino acids (BCAA) have shown to individually improve body composition and metabolic rate in overweight and obese individuals. The purpose of this study was to investigate the effects of a multi-ingredient dietary supplement (MIDS) containing these ingredients on body composition, lipid profile, and metabolic rate in overweight and obese individuals.

## Methods

Forty-nine healthy, sedentary, overweight or obese men and women were stratified by body fat percentage and randomly assigned to two groups: 1) a soybean oil placebo (PL) or 2) a MIDS containing 500 mg of green tea extract (45% EGCG), 99 mg of caffeine, and a proprietary blend containing 1260 mg of CLA, L-leucine, L-isoleucine and L-valine per serving. Twenty-nine participants completed the study (Mean ± SD; PL: n=16; age, 27.7 + 10.6 yrs; body mass, 88.7 + 3.7 kg; BMI, 31.5 ± 4.6; body fat% 42.3 + 7.2; MIDS n=13; age, 31.8 + 11.3 yrs; body mass, 95.5 + 4.1 kg; BMI, 33.5 + 4.2; body fat% 44.5 + 6.1) with 14 participants withdrawing due to personal reasons or time constraints and 6 people excluded due to low compliance (<80%). Both groups consumed one serving with breakfast and one serving with lunch for 8 weeks with no other changes to nutrition or exercise habits. Laboratory testing took place at baseline and after the 8-week intervention. Body composition was analyzed with dual-energy x-ray absorptiometry. Resting metabolic rate (RMR), lipid profile, waist and hip circumferences were measured while subjects were fasting. Data were analyzed using JMP 09 Pro (Cary, NC), with alpha level at 0.05. A one-way ANOVA was used to evaluate baseline differences and a two-way ANOVA with repeated measurements was used to evaluate changes in dependent variables over time. If significant interactions were found, a Tukey test was used for post hoc comparisons.

## Results

No significant changes were measured for body mass (BM) or lean body mass (LBM) in either group. A group x time effect for total body fat percent (P=0.01; mean ± SE; PL baseline, 42.3 ± 0.2% to post, 42.6 ± 0.2%,+ 0.71 %; MIDS baseline, 44.5 ± 0.2% to post, 43.8 ± 0.2%,-2.24%) and android fat percent (P= 0.03; PL baseline, 49.1 ± 0.2% to post, 49.3 ± 0.2%,+ 0.4%; MIDS baseline, 51.8 ± 0.3 % to post, 50.9 ± 0.3%, - 0.9%) was observed. There was a main time effect where satiety increased (P= 0.004) and desire to eat decreased (P=0.007). No other changes were reported. The side effects reported with MIDS were headache (n=1), anxiety (n=1), and jitteriness (n=1) and for PL were headache (n=1), bloated feelings (n=1), and improved bowel movements (n=1).

## Conclusion

Eight weeks of MIDS supplementation significantly decreased total and android fat percent. A main time effect was observed for satiety and desire to eat. Health indices of blood pressure, heart rate and blood lipids did not differ between groups.

